# Selective Effects of PDE10A Inhibitors on Striatopallidal Neurons Require Phosphatase Inhibition by DARPP-32^1^,^2^,^3^

**DOI:** 10.1523/ENEURO.0060-15.2015

**Published:** 2015-08-31

**Authors:** Marina Polito, Elvire Guiot, Giuseppe Gangarossa, Sophie Longueville, Mohamed Doulazmi, Emmanuel Valjent, Denis Hervé, Jean-Antoine Girault, Danièle Paupardin-Tritsch, Liliana R. V. Castro, Pierre Vincent

**Affiliations:** 1 CNRS, UMR8256 “Biological Adaptation and Ageing”, Institut de Biologie Paris-Seine (IBPS), F-75005 Paris, France; 2 Université Pierre et Marie Curie (UPMC, Paris 6), Sorbonne Universités, Paris, F-75005, France; 3 CNRS, UMR-5203, Institut de Génomique Fonctionnelle, Montpellier, F-34094, France; 4 Institut National de la Santé et de la Recherche Médicale, U661, Montpellier, F-34094, France; 5 Universités de Montpellier 1 & 2, UMR-5203, Montpellier, F-34094, France; 6 Institut National de la Santé et de la Recherche Médicale UMR-S 839, Paris, France; 7 Institut du Fer à Moulin, Paris, France

**Keywords:** biosensor imaging, cAMP, phosphodiesterase, protein kinase, schizophrenia, striatum

## Abstract

Type 10A phosphodiesterase (PDE10A) is highly expressed in the striatum, in striatonigral and striatopallidal medium-sized spiny neurons (MSNs), which express D_1_ and D_2_ dopamine receptors, respectively. PDE10A inhibitors have pharmacological and behavioral effects suggesting an antipsychotic profile, but the cellular bases of these effects are unclear. We analyzed the effects of PDE10A inhibition *in vivo* by immunohistochemistry, and imaged cAMP, cAMP-dependent protein kinase A (PKA), and cGMP signals with biosensors in mouse brain slices. PDE10A inhibition in mouse striatal slices produced a steady-state increase in intracellular cAMP concentration in D_1_ and D_2_ MSNs, demonstrating that PDE10A regulates basal cAMP levels. Surprisingly, the PKA-dependent AKAR3 phosphorylation signal was strong in D_2_ MSNs, whereas D_1_ MSNs remained unresponsive. This effect was also observed in adult mice *in vivo* since PDE10A inhibition increased phospho-histone H3 immunoreactivity selectively in D_2_ MSNs in the dorsomedial striatum. The PKA-dependent effects in D_2_ MSNs were prevented in brain slices and *in vivo* by mutation of the PKA-regulated phosphorylation site of 32 kDa dopamine- and cAMP-regulated phosphoprotein (DARPP-32), which is required for protein phosphatase-1 inhibition. These data highlight differences in the integration of the cAMP signal in D_1_ and D_2_ MSNs, resulting from stronger inhibition of protein phosphatase-1 by DARPP-32 in D_2_ MSNs than in D_1_ MSNs. This study shows that PDE10A inhibitors share with antipsychotic medications the property of activating preferentially PKA-dependent signaling in D_2_ MSNs.

## Significance Statement

The striatum is mainly composed of medium-sized spiny neurons that express either dopamine D_1_ receptors or dopamine D_2_ receptors. Their activity is associated with either the initiation of movement or action suppression, respectively. Biosensor imaging revealed that pharmacological inhibition of type 10A phosphodiesterase increased cAMP levels in D_1_ and D_2_ neurons in the same manner, but only D_2_ neurons exhibited an increase in the protein kinase A-mediated phosphorylation level. This effect resulted from an asymmetrical regulation of phosphatases by DARPP-32. D_2_ neurons are thus more prone to respond to a tonic cAMP signal than D_1_ neurons, a property that may explain how phosphodiesterase 10A inhibitors produced antipsychotic-like behavioral effects. This D_1_/D_2_ imbalance may also be critical for reward-mediated learning and action selection.

## Introduction

Schizophrenia is a devastating psychiatric disease, which results in persistent cognitive and emotional impairments. Type 10A phosphodiesterase (PDE10A) inhibitors were recently proposed as a treatment for schizophrenia (Kehler and Nielsen, 2011; Chappie et al., 2012); however, their cellular mechanisms of action remain unclear with respect to their putative therapeutic effects. PDE10A is highly and almost exclusively expressed in medium-sized spiny neurons (MSNs) of the striatum (Seeger et al., 2003; Coskran et al., 2006; Heiman et al., 2008; Lakics et al., 2010; Kelly et al., 2014). MSNs are divided into two categories based on their expression of dopamine receptors and their sites of projection, as follows: MSNs projecting to the substantia nigra highly express dopamine D_1_ receptors (hereafter termed D_1_ MSNs); whereas, MSNs projecting to the external globus pallidus highly express adenosine A_2A_ and dopamine D_2_ receptors (hereafter termed D_2_ MSNs; Gerfen et al., 1990; Le Moine and Bloch, 1995; Bertran-Gonzalez et al., 2008; Matamales et al., 2009). The high expression of PDE10A in MSNs, and its interaction with the scaffold protein AKAP150 (A-kinase anchoring protein 150), protein kinase A (PKA), PSD-95, and NMDA receptor suggests an important role in modulating the spread of the synaptic cAMP signals into the cell body (Russwurm et al., 2015).

Besides PDE10A, striatal neurons express a number of specific signaling proteins that markedly differ from those in other brain regions (Girault, 2012), and that determine the characteristics of the cAMP/PKA signaling pathway (Castro et al., 2013). Among these specific proteins, DARPP-32 (32-kDa dopamine and cAMP-regulated phosphoprotein) is a multifunctional protein regulating phosphatase and kinase activities: for example, when DARPP-32 is phosphorylated at threonine 34 residue (Thr34) by PKA, it becomes a potent inhibitor of serine/threonine protein phosphatase-1 (PP-1; Hemmings et al., 1984; Svenningsson et al., 2004), increasing the duration of PKA-dependent signals (Castro et al., 2013).

Classical and atypical antipsychotic agents share the property of inhibiting D_2_ receptors and thus, in the striatum, increase PKA-dependent phosphorylation selectively in D_2_ MSNs (Bateup et al., 2008; Bertran-Gonzalez et al., 2008, 2009). In contrast, psychostimulants, which are psychotomimetic, activate many signaling responses in D_1_ MSNs (Bateup et al., 2008; Bertran-Gonzalez et al., 2008, 2009). PDE10A inhibitors were shown to increase cAMP levels in the striatum (Schmidt et al., 2008) and could be expected to mimic the effects of both antipsychotic and psychotomimetic compounds. We used biosensor imaging to precisely analyze the effects of PDE10A inhibitors on cAMP/PKA signaling at the level of individual D_1_ and D_2_ MSNs. Our work revealed that although PDE10A inhibition increased intracellular cAMP levels in both D_1_ and D_2_ MSNs, the downstream consequences at the level of PKA targets were profoundly different: the cAMP signal resulting from PDE10A inhibition strongly increased PKA-dependent phosphorylation in D_2_ MSNs, whereas D_1_ MSNs remained mostly unaffected. Further analyses showed that the difference required DARPP-32-dependent regulation of phosphatase activity in D_1_ and D_2_ MSNs.

## Materials and Methods

### Animals

Animals were housed under standardized conditions with a 12 h light/dark cycle, stable temperature (22 ± 1ºC), controlled humidity (55 ± 10%), and food and water available ad libitum. Homozygous mice expressing DARPP-32 with the T34A or T75A mutation (Svenningsson et al., 2003) were obtained by crossing heterozygous mice, on a mixed C57BL6/J-Sv129 background (a gift of Dr. P. Greengard, The Rockefeller University, New York). Male Drd2-EGFP heterozygous mice (C57Bl6/J) were generated as described previously (Gong et al., 2003). Experiments were performed in accordance with the regulations under the control of the local ethic committee Charles Darwin C2EA - 05.

### Live brain slice preparation

Brain slices were prepared from male mice that were 8–12 days of age. Coronal brain slices were cut with a VT1200S microtome. Slices were prepared in an ice-cold solution of the following composition (in mm): 125 NaCl, 0.4 CaCl_2_, 1 MgCl_2_, 1.25 NaH_2_PO_4_, 26 NaHCO_3_, 25 glucose, and 1 kynurenic acid, saturated with 5% CO_2_ and 95% O_2_. The slices were incubated in this solution for 30 min and then placed on a Millicell-CM membrane (Millipore) in culture medium (50% Minimum Essential Medium, 50% HBSS, 6.5 g/L glucose, penicillin-streptomycin; Invitrogen). We used the Sindbis virus as a vector to induce expression of the various biosensors after overnight incubation (Ehrengruber et al., 1999). The coding sequences of Epac-S^H150^ (Polito et al., 2013), AKAR2-NLS (Zhang et al., 2005), AKAR3 (Allen and Zhang, 2006), and cygnet2 (Honda et al., 2001) were inserted into the viral vector pSinRep5 (Invitrogen). The viral vector (∼5 × 10^5^ particles per slice) was added, and slices were incubated overnight at 35°C under an atmosphere containing 5% CO_2_. Before the experiment, slices were incubated for 30 min in the recording solution (125 mm NaCl, 2 mm CaCl_2_, 1 mm MgCl_2_, 1.25 mm NaH_2_PO_4_, 26 mm NaHCO_3_, and 25 mm glucose, saturated with 5% CO_2_ and 95% O_2_). Recordings were performed with a continuous perfusion of the same solution at 32°C. MSNs constitute 95% of neurons in the striatum. Large neurons (smallest soma diameter, >14 µm), presumably cholinergic interneurons, were excluded.

### Live brain slice imaging

For two-photon imaging, excitation was obtained using a Ti:sapphire laser (MaiTai HP; Spectra Physics) tuned at 850 nm for CFP excitation. Galvanometric scanners (model 6210; Cambridge Technology) were used for raster scanning, and a piezo-driven objective scanner (P-721 PIFOC; Physik Instrumente GmbH) was used for *z*-stack image acquisition. The system was controlled by MPscope software (Nguyen et al., 2006). The microscope was based on an Olympus BX51WI upright microscope with a 40× 0.8 numerical aperture (NA) or 60× 0.9 NA water-immersion objective. A two-photon emission filter was used to reject residual excitation light (E700 SP; Chroma Technology). A fluorescence cube containing 479/40 and 542/50 emission filters and a 506 nm dichroic beamsplitter (FF01-479/40, FF01-542/50 and FF506-Di02-25x36 Brightline Filters; Semrock) was used for the orthogonal separation of the two fluorescence signals. Two imaging channels (H9305 photomultipliers; Hamamatsu) were used for simultaneous detection of the two types of fluorescence emission. For each data point, an image stack of 30–40 images with a 0.5 µm interval was acquired.

Wide-field images were obtained with an Olympus BX50WI or BX51WI upright microscope with a 40× 0.8 NA water-immersion objective and an ORCA-AG Camera (Hamamatsu). Images were acquired with iVision (Biovision). The excitation and dichroic filters were D436/20 and 455dcxt. Signals were acquired by alternating the emission filters, HQ480/40 for CFP, and D535/40 for yellow fluorescent protein, with a filter wheel (Sutter Instruments). These filters were obtained from Chroma Technology.

No correction for bleed-through or direct excitation of the acceptor was applied, since this correction, while increasing the absolute amplitude of ratio changes, also increases the noise in the measurement (Ducros et al., 2009).

The biosensor chromophores are sensitive to nonspecific environmental disturbances. We used a mutated version of AKAR3 in which the threonine residue of the PKA phosphorylation site was replaced with an alanine residue (T391A) as a control. This AKAR3 (T391A) control sensor reported no ratio change in response to PDE10A inhibition in MSNs.

### Data analysis

Images were analyzed with custom routines written in the IGOR Pro environment (Wavemetrics). The emission ratio was calculated for each pixel, as follows: F535/F480 for AKAR2-NLS and AKAR3, and F480/F535 for cygnet2 and Epac-S^H150^ sensors. The pseudocolor images display the ratio value coded in hue and the fluorescence of the preparation coded in intensity.

**Figure 1.**
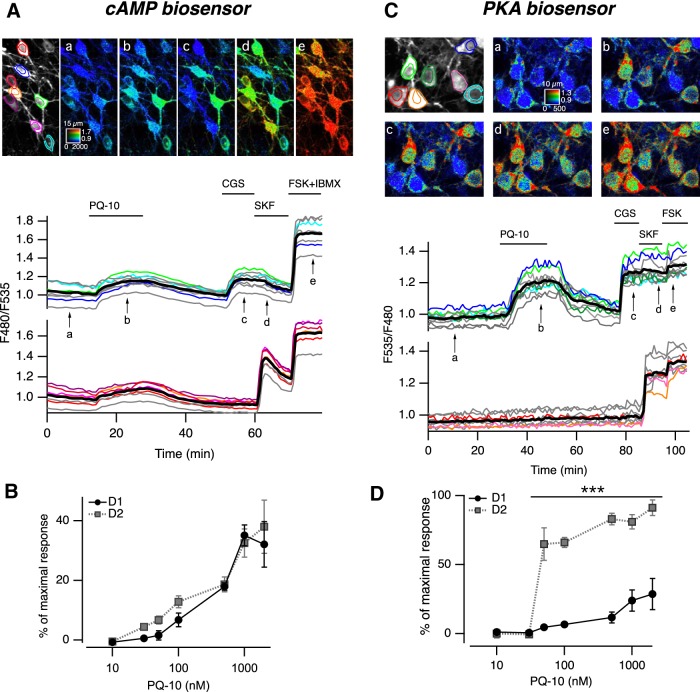
PDE10A inhibition increases cAMP levels in both in D_1_ and D_2_ MSNs, and PKA-dependent phosphorylation only in D_2_ MSNs. ***A***, MSNs in **a** neostriatal mouse brain slice expressing the cAMP biosensor Epac-S^H150^ were imaged with two-photon microscopy during the application of PQ-10 (100 nm). Images (vertical projection of the image stack) show the raw fluorescence at 535 nm (left, in grayscale) and the ratio (in pseudocolor) indicating intracellular cAMP concentrations, at the times indicated by the arrows on the graph below. The calibration square in ***A*** indicates the spatial scale (the size of the square is indicated in micrometers), and shows the ranges of intensity (horizontally) and ratio (vertically). Each trace on the graph indicates the F480/F535 emission ratio measured in regions indicated by the color contour drawn on the raw image. Traces in gray correspond to regions that are not visible on these images. Traces are plotted in two groups according to their response to either CGS 21680, an adenosine A_2A_ receptor agonist (CGS, 1 µm), or SKF-38393, a D_1_-like receptor agonist (SKF, 1 µm). The thick black line represents the average of all the traces in a group. FSK **(**13 µm) and IBMX (200 µm) were applied at the end of the recording to determine the maximal response. ***B***, The same experiment was repeated for every PQ-10 concentration tested. No significant difference was found between D_1_ and D_2_ MSNs (two-way ANOVA: dose effect, F_(6,54)_ = 40.91, *p* < 10**^−^** ^4^; D_1_/D_2_ effect, F_(1,54)_ = 2.56, *p* = 0.115; dose × D_1_/D_2_ interaction, *F*_(6,54)_ = 0.625, *p* = 0.709**)**. Error bars indicate the SEM. ***C*,** Same as ***A***, except that the AKAR3 biosensor was used to monitor PKA-dependent phosphorylation, and the ratio was calculated as F535/F480. ***D***, Same as ***B*** for AKAR3 measurements. Data were analyzed with two-way ANOVA: dose effect, *F*_(6,38)_ = 28.31, *p* < 10**^−^** ^4^; D_1_/D_2_ effect, *F*_(1,38)_ = 143.73, *p* < 10**^−^** ^4^; dose × D_1_/D_2_ interaction, *F*_(6,38)_ = 9.23, *p* < 10**^−^** ^4^ Bonferroni’s *post hoc* test, ***p < 0.001 .

**Figure 2.**
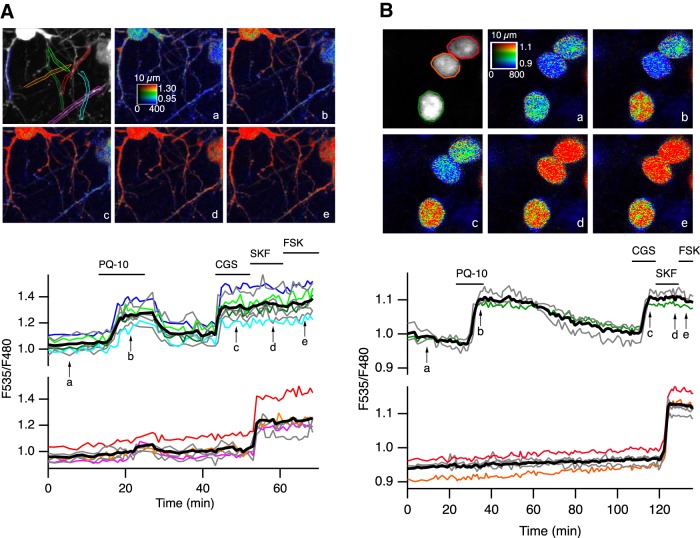
PDE10A inhibition triggers positive PKA responses in dendrites and nuclei preferentially in D_2_ MSNs. ***A***, ***B*,** Brain slices expressed the PKA sensor AKAR3 (***A***) or AKAR2-NLS (***B***) and were imaged by two-photon microscopy during the application of PQ-10 (100 nm). Images show the raw fluorescence at 535 nm (left in grayscale) and the ratio (in pseudocolor) indicating the PKA**-**dependent phosphorylation level of the biosensor, at the times indicated by the arrows on the graph below. The calibration square in ***A*** indicates the spatial scale (above, in micrometers), and shows the ranges of intensity (horizontally) and ratio (vertically). Each trace on the graph indicates the F535/F480 emission ratio measured on regions indicated by the color contour drawn on the raw image. Traces are plotted in two groups according to their response to either CGS 21680 (CGS, 1 µm) or SKF-38393 (SKF, 1 µm). The thick black line represents the average of all the traces in a group. FSK (13 µm) was applied at the end of the recording to determine the maximal response.

**Figure 3.**
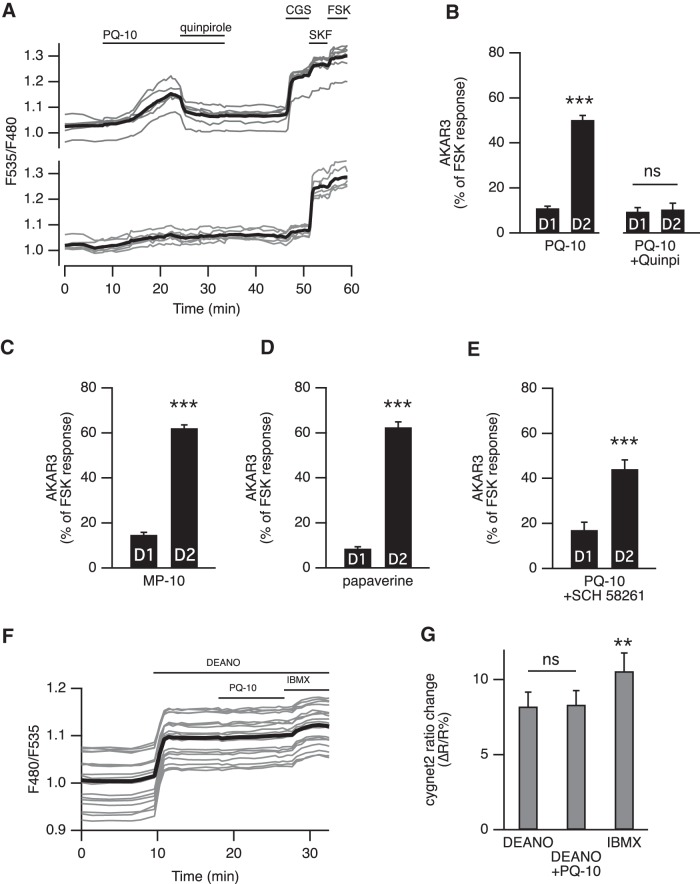
***A***, Activation of D_2_ dopamine receptors suppressed the effect of PDE10A inhibition on AKAR3 ratio. Each trace on the graph indicates the ratio measurement on MSNs expressing AKAR3 and identified as D_1_ or D_2_ according to their response to either SKF-38393 (SKF, 1 µm) or CGS 21680 (CGS, 1 µm), respectively. The thick black line represents the average of all the traces in a group. Bath application of the agonist of dopamine D_2_ receptors quinpirole (1 µm) reversed the response to PQ-10 (100 nm). ***B***, D_2_ receptor activation prevented the response to PDE10A inhibition: the effect of PQ-10 was measured in the presence of the D_2_ agonist quinpirole (1 µm). No statistically significant difference (p > 0.05) was found between D_1_ and D_2_ MSNs (*n* = 5). The effect of PQ-10 is displayed for comparison on the left (same data as in Fig. 4E). ***C***, ***D***, Other PDE10A inhibitors also increased the AKAR3 ratio preferentially in D_2_ MSNs: MP-10 (***C***; 100 nm, *n* = 9) and papaverine (***D***; 1 µm, *n* = 5) both increased **the** AKAR3 ratio selectively in D_2_ MSNs. ***E***, PQ-10 increased AKAR3 ratio selectively in D_2_ MSNS even when adenosine A_2A_ receptors were inhibited with 100 µm SCH 58261 (*n* = 4). ***B–E***, Statistical differences were tested with paired Student’s *t* test. ***p < 0.001. ***F***, PDE10A inhibition had no effect on cGMP levels measured with the cGMP sensor cygnet2. The NO donor DEANO (100 µm) increased the ratio; after reaching a steady-state level, PQ-10 (1 µm) was added; at the end of the recording, the maximal ratio response was elicited by DEANO plus IBMX (200 µm). ***G***, No difference was measured when comparing the response with DEANO alone and DEANO with PQ-10, while IBMX produced a significant increase. The data expressed as the mean ± SEM were analyzed by repeated-measures one**-**way ANOVA F_(1_**_,_**_5)_ = 11,224, *p* < 0.001, *n* = 6, followed by Bonferroni’s *post hoc* test: **p < 0.01)**. *A–G***, Brain slices were imaged with wide-field microscopy. All data are expressed as the mean ± SEM.

Two-photon imaging was used to separate individual neurons for a precise quantification of the amplitude of the response (Figs. 1, 2). Ratio measurements were performed on a series of 5–10 consecutive image from the image stack, centered on the cell body. With cytosolic biosensors, when visible, the nucleus was excluded from the measurement. Wide-field imaging (Figs. 3*A*–E) also allowed the unambiguous identification of D_1_ and D_2_ MSNs, provided that the infection level was kept low and no fluorescence overlap between neighboring neurons was observed. The optical cross-contamination resulting from out-of-focus light was evaluated by the final response to CGS 21680 and SKF-38393, applied sequentially: cells were rejected from analysis if the cross-contamination was >30%. For cGMP imaging (Fig. 3*F*,*G*), the data were quantified as relative ratio change.

### Quantifications of cAMP signals

The amplitudes of responses were quantified for each neuron as the fractional change in ratio from its own baseline and maximal final ratio response. Responses obtained from MSNs of the same type were averaged for each experiment (i.e., brain slice).

The free cAMP concentrations were estimated with the Epac-S^H150^ biosensor from the ratio measurement using the Hill equation, with a *K*_d_ of 4.4 µm and a Hill coefficient of 0.77, as determined from Polito et al. (2013). The maximal response corresponding to biosensor saturation (*R*_max_) was determined for each neuron at the end of the recording. This level was obtained by applying 13 µm forskolin (FSK), a dose known to be sufficient to maximally phosphorylate the highly sensitive probe AKAR3 in MSNs. For cAMP biosensors, this *R*_max_ value was obtained with 200 µm IBMX and 13 µm forskolin.

The baseline cAMP level in control conditions was evaluated by inhibiting adenylyl cyclases with 200 µm SQ22536, which resulted in a ratio decrease, measured in wide-field microscopy, of −4.0% of the maximal ratio change. This decrease in baseline ratio was −4.9 ± 0.7 and −3.3 ± 0.7 (*n* = 6, p < 0.05 with paired Student’s *t* test), respectively, in D_1_ and D_2_ MSNs. Assuming that adenylyl cyclase inhibition effectively decreased cAMP levels down to a level sufficient to reach the minimal ratio level (*R*_min_), these values suggest a baseline cAMP concentration in a range of ∼100 nm.

### Tissue preparation and immunofluorescence

Mice, 8-10 weeks old, were treated with the drug for 60 min and then rapidly anesthetized with pentobarbital (500 mg/kg, i.p.; Sanofi-Aventis) and were transcardially perfused with 4% (w/v) paraformaldehyde in 0.1 m PBS, pH 7.5. Brains were post-fixed overnight in the same solution and stored at 4°C. The 30-μm-thick sections were cut with a vibratome and stored at −20°C in a solution containing 30% (v/v) ethylene glycol, 30% (v/v) glycerol and 0.1 m sodium phosphate buffer, until they were processed for immunofluorescence. Sections were processed as described in Bertran-Gonzalez et al. (2009). Sodium fluoride 0.1 mm was included in all buffers and incubation solutions. Histone H3 phosphorylation was revealed with a rabbit polyclonal antibody against phospho-Ser10-H3 (1:1000; catalog #06570; Millipore) the specificity of which was confirmed in a previous study (Jordi et al., 2013). GFP was detected using chicken antibody against GFP (1:500; catalog #A10262; Life Technologies). Following incubation with primary antibodies, sections were rinsed three times for 10 min in TBS and incubated for 45–60 min with goat Cy3-coupled (1:500; Jackson ImmunoResearch; Fig. 5) and goat A488 (1:500; Life Technologies; Fig. 6). Sections were rinsed for 10 min twice in Tris-buffered saline and twice in Tris buffer (0.25 m Tris) before mounting in 1,4-diazabicyclo-[2. 2. 2]-octane (Sigma-Aldrich).

Single-labeled images (Fig. 6) were obtained with a Zeiss LSM780 Confocal Microscope. Double-labeled images (Fig. 5) were obtained with a Leica TCS SPE Confocal Microscope with laser lines at 496 and 561 nm, acquiring in the 501–539 nm and 570–660 nm bands. All parameters were held constant for all sections from the same experiment.

### Statistics

Data were analyzed with SPSS statistical software version 22.0. Normality in variable distributions and homogeneity of variances across groups were assessed with the Shapiro–Wilk and Levene tests, respectively. Variables that failed any of these tests were analyzed with nonparametric statistics using the Kruskal–Wallis ANOVA on ranks followed by Mann–Whitney rank sum test with a Dunn–Sidak adjustment test for pairwise multiple comparisons. Variables that passed the normality test were analyzed with ANOVA followed by Bonferroni post hoc test for multiple comparisons or by Student’s *t* test for comparing two groups. Paired data were analyzed with a Student’s *t* test. A *p* value of <0.05 was used as a cutoff for statistical significance. All error bars represent the SEM; *n* indicates the number of experiments (i.e., the number of brain slices tested), with at least four neurons of each type in each experiment. All experiments were performed on at least three different brain slices from at least two animals.

### Drugs

SKF-38393 hydrobromide, CGS 21680 hydrochloride, 1-methyl-3-isobutylxanthine (IBMX), rolipram, papaverine, roscovitine, okadaic acid, cantharidin, gabazine, CNQX, APV, and forskolin were obtained from Tocris Cookson. TTX was from Latoxan. PQ-10, MP-10, and roflumilast were a gift from Janssen Pharmaceuticals. TP-10 was provided by Pfizer through the Compound Transfer Program.

## Results

### PDE10A inhibition reveals a tonic cAMP production in both D_1_ and D_2_ MSNs

Since both D_1_ and D_2_ MSNs express high levels of PDE10A protein (Nishi et al., 2008), we used biosensor-imaging approaches to compare the effects of PDE10A inhibition on the cAMP/PKA signaling cascade in D_1_ and D_2_ MSNs. First, we monitored changes in intracellular cAMP concentrations with two-photon microscopy in striatal brain slices expressing Epac-S^H150^. At the end of every experiment, an agonist of adenosine A_2A_ receptors (CGS 21680, 1 µm) and an agonist of dopamine D_1_ receptors (SKF-38393, 1 µm) were applied sequentially, triggering a positive cAMP response in D_2_ and D_1_ MSNs, respectively, thereby functionally identifying MSN subtypes. The final application of the general adenylyl cyclase activator FSK (13 µm) together with the nonselective phosphodiesterase inhibitor IBMX (200 µm) produced the maximal ratio response used for normalization.

PDE10A inhibition with PQ-10 (100 nm) increased cAMP levels in all MSNs (Fig. 1*A*) in a dose-dependent manner. This dose dependency was not statistically different between D_1_ and D_2_ MSNs (Fig. 1*B*). At the highest doses (1 and 2 µm), cAMP responses reached ∼35% of the maximal response to FSK plus IBMX, which corresponds to a concentration of free cAMP of ∼2 µm (see Materials and Methods for details on the estimation of cAMP concentrations). These experiments showed that, in the basal condition, cAMP is tonically produced in striatal slices and that PDE10A contributes significantly to its degradation.

### PDE10A inhibition increases the phosphorylation of a PKA target exclusively in D_2_ MSNs

We then analyzed the effects of PQ-10 on PKA-dependent phosphorylation levels using the PKA biosensor AKAR3. As for cAMP imaging, MSNs were identified at the end of each experiment by their response to either A_2A_ or D_1_ receptor agonist. The maximal AKAR3 response was elicited by FSK. Although the increase in free cAMP concentration was similar in D_1_ and D_2_ MSNs (Fig. 1*A*,*B*), the resulting PKA-dependent phosphorylation levels were completely different (Fig. 1*C*): PQ-10 (100 nm) strongly increased the emission ratio of AKAR3 in the D_2_ MSNs (66 ± 4% of the maximal response to FSK, *n* = 4); whereas, in D_1_ MSNs, the ratio remained at a much lower level (6 ± 2%). These results indicated a significantly higher phosphorylation of AKAR3 in D_2_ than in D_1_ MSNs in response to PQ-10. The effect of PQ-10 on AKAR3 ratio in D_2_ MSNs was steeply dose dependent with a maximal effect reached at <100 nm (Fig. 1*D*). In stark contrast to D_2_ MSNs, even high doses of PQ-10, which increased cAMP to the same levels in D_1_ and D_2_ MSNs (Fig. 1*B*), only produced a very small effect on AKAR3 phosphorylation in D_1_ MSNs (Fig. 1*D*). These experiments thus revealed a much stronger effect of cAMP on PKA-dependent phosphorylation in D_2_ than in D_1_ MSNs.

PDE10A may be addressed differentially in the cytoplasm and membranes (Kotera et al., 2004; Charych et al., 2010), and cAMP dynamics could differ in subcellular domains of different geometry, like dendrites (Castro et al., 2010). An increase in AKAR3 ratio was observed exclusively in the dendritic branches that responded to the A_2A_ agonist, whereas dendrites, which responded to the D_1_ agonist, showed no response to 100 nm PQ-10 (Fig. 2*A*). This is consistent with other biosensor recordings in which dendrites of D_1_ MSNs also exhibited no baseline response to PDE10A inhibition (Yagishita et al., 2014).

Once activated, PKA can translocate to the nucleus and phosphorylate a number of nuclear proteins. We examined whether the differential response to PQ-10 also existed in the nucleus. Using the nuclear AKAR2-NLS biosensor, we found that PDE10A inhibition induced a strong ratio increase in D_2_ MSNs, while D_1_ MSNs remained unresponsive (Fig. 2*B*). These results showed that PQ-10 efficiently increased AKAR3 phosphorylation in the cytoplasm and nucleus of D_2_ but not D_1_ MSNs, whereas they had a similar and selective effect on cAMP production in the two cell types.

### PDE10A inhibition effects are antagonized by D_2_ receptors and independent of A_2A_ receptors

One feature of striatopallidal MSNs is the coexpression of D_2_ dopamine and A_2A_ adenosine receptors, negatively and positively coupled to adenylyl cyclase, respectively (Schiffmann et al., 1991; Schiffmann and Vanderhaeghen, 1993; Ferré et al., 1997; Svenningsson et al., 1999; Bateup et al., 2008; Bertran-Gonzalez et al., 2009). Application of the D_2_ receptor agonist quinpirole (1 µm) completely reversed the PQ-10-induced AKAR3 response in D_2_ MSNs, monitored with wide-field microscopy (Fig. 3*A*). Application of quinpirole alone had no effect on the basal AKAR3 ratio but prevented positive responses to PQ-10 in D_2_ MSNs with 11 ± 3% (*n* = 5) of the maximal FSK response in D_2_ MSNs compared with 9 ± 2% in D_1_ MSNs (Fig. 3*B*). These results showed that the activation of D_2_ receptors opposed the effect of PQ-10 in D_2_ MSNs, most likely via Gi-mediated inhibition of the tonic adenylyl cyclase activity. These experiments also further confirmed that the positive AKAR3 response to PQ-10 was specific to MSNs expressing D_2_ receptors.

We then determined whether the effect selectivity for D_2_ MSNs was a particular property of PQ-10 or was also observed with other PDE10A inhibitors. The effects of MP-10 (100 nm) and papaverine (1 µm) on AKAR3 ratio were similar (Fig. 3*C*,*D*), inducing an AKAR3 ratio increase selectively in D_2_ MSNs. TP-10 also produced the same response profile (see below). In contrast, PDE4 inhibitors (rolipram, 100 nm, *n* = 4; and roflumilast, 1 µm, *n* = 4) had no effect on basal AKAR3 ratio (data not shown).

As D_2_ MSNs express adenosine A_2A_ receptors, we examined whether the tonic presence of extracellular adenosine in our brain slice preparation activated adenylyl cyclase and might be responsible for the positive response to PQ-10 recorded specifically in these MSNs. When A_2A_ receptors were blocked with SCH 58261 (100 nm), PQ-10 still elicited positive responses in D_2_ MSNs (Fig. 3*E*). In contrast, SCH 58261 blocked the responses to the A_2A_ agonist CGS 21680 (data not shown). These results showed that tonic activation of A_2A_ receptors was not required for the phosphorylation response to PQ-10 in D_2_ MSNs. We then examined whether the phosphorylation signal involved endogenous neuronal activity, or required the glutamate and GABA which may be present in the brain slice. This was not the case since, in the presence of blockers of voltage-gated sodium channels (TTX, 100 nm), calcium channels (CdCl_2_, 200 µm), non-NMDA receptors (CNQX, 10 µm), NMDA receptors (APV, 10 µm), and GABA_A_ receptors (SR 95531, 1 µm), the selective effect of PQ-10 on D_2_ MSNs was still present (25 ± 3 vs 71 ± 10 of the maximal FSK response in D_1_ and D_2_ MSNs, respectively; *n* = 3, paired Student’s *t* test; data not shown).

Since PDE10A also degrades cGMP and PDE10A inhibition was shown to increase cGMP levels and affect synaptic transmission *in vivo* (Siuciak et al., 2006; Schmidt et al., 2008; Grauer et al., 2009; Padovan-Neto et al., 2015), we used wide-field imaging of the cGMP biosensor cygnet2 (Honda et al., 2001) to determine whether PDE10A also regulated cGMP in MSNs. PQ-10 (1 µm) had no effect on the baseline cGMP levels (*n* = 4, *p* = 0.44, one-sample Student’s *t* test). In addition, PQ-10 (1 µm) had no effect on the cGMP steady-state level obtained with the nitric oxide (NO) donor DEANO (Diethylamine nitric oxide, 100 µm; *n* = 6; Fig. 3*F*), while the nonspecific phosphodiesterase inhibitor IBMX produced a significant ratio increase. These results indicated that in our conditions PDE10A inhibition does not significantly affect cGMP levels in MSNs.

### Different responsiveness of D_1_ and D_2_ MSNs is abolished by protein phosphatase inhibition

The difference between D_1_ and D_2_ MSNs in the phosphorylation level of AKAR3 could result from differences in the rate of phosphorylation by PKA, dephosphorylation by phosphatases, or both. A difference in PKA levels is unlikely because immunostaining of catalytic subunits in the striatum did not reveal major differences between cells (Yang et al., 2014). Since AKAR3 biosensor responses to PKA activation are reversed by the action of endogenous protein phosphatases (Gervasi et al., 2007), we investigated the role of protein phosphatases in the different responsiveness of D_1_ and D_2_ MSNs to PDE10A inhibitors.

**Figure 4.**
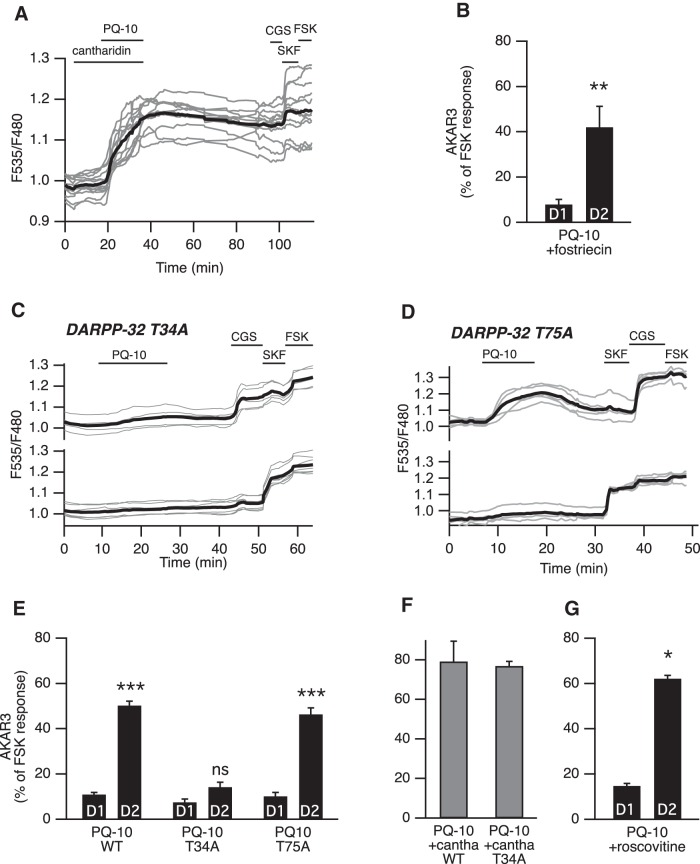
DARPP-32-mediated phosphatase inhibition favors PKA signaling in D_2_ MSNs. ***A***, PP-1 and PP-2A were inhibited with cantharidin. Cantharidin (30 µm) alone did not change the basal ratio but strongly increased the AKAR3 response to PQ-10 (100 nm) in all MSNs. These responses were not reversible, making the final identification of D_1_ and D_2_ MSNs impossible (gray bars in ***F***, which represent the responses of all MSNs). ***B***, D_2_ MSNs responded selectively to PQ-10 (100 nm) even when the PP-2A inhibitor fostriecin (200 nm) was applied (*n* = 4, paired Student’s *t* test; **p < 0.01). ***C–E***, Mutation of the Thr34 to Ala in DARPP-32 (DARPP-32 T34A) strongly reduced the effect of PQ-10 (100 nm) in D_2_ MSNs, whereas the selective effect of PQ-10 on D_2_ MSNs remained in brain slices from animals bearing the Thr75 to Ala mutation in DARPP-32 (DARPP-32 T75A). ***C*, *D***, Representative experiments performed with DARPP-32 T34A (***C***) and DARPP-32 T75A (***D***) knock-in mice. Each trace on the graph indicates the ratio measurement on MSNs expressing AKAR3 and is identified as D_1_ or D_2_ according to their response to either SKF-38393 (SKF, 1 µm) or CGS 21680 (CGS, 1 µm), respectively. The thick black line represents the average of all the traces in each group. ***E***, The data expressed as the mean ± SEM were analyzed by two-way ANOVA: genotype effect, F_(2,72)_ = 71.12, *p* < 10**^−^** ^4^; D_1_/D_2_ effect, F_(1,72)_ = 333.07, *p* < 10**^−^** ^4^; genotype **×** D_1_/D_2_ interaction, F_(2, 72)_ = 49.53, *p* < 10**^−^** ^4^. Bonferroni’s *post hoc* test: ***p < 0.001. ***F***, In wild-type (WT) mice and DARPP-32 T34A mutants, and in the presence of cantharidin (30 µm), all MSNs responded to PQ-10 (100 nm) with an increase in AKAR3 ratio such that D_1_ and D_2_ MSNs could not be distinguished (*n* = 5 for both). No significant difference was obtained between wild-type and DARPP-32 T34A mutant (unpaired Student's *t* test, p > 0.05). ***G***, D_2_ MSNs responded selectively to PQ-10 (100 nm) even when the Cdk5 inhibitor roscovitine (10 µm) was applied (*n* = 4, paired Student’s *t* test; *p < 0.05).

Cantharidin (30 µm), a nonselective inhibitor of PP-1 and PP-2A, had no effect by itself on the AKAR3 emission ratio (Fig. 4*A*). However, when PQ-10 (100 nm) was applied in the bath (Fig. 4*A*), the AKAR3 ratio increased in virtually all D_1_ and D_2_ MSNs (79 ± 10% of the FSK response, *n* = 5). Since these responses did not return to the baseline after drug washout, it was impossible to distinguish between D_1_ and D_2_ MSNs, and all MSNs were pooled (Fig. 4*F*, gray color bar). To identify which phosphatase subtype was involved in the response, we used fostriecin (200 nm), a selective inhibitor of PP-2A (Swingle et al., 2009). We observed no effect of this inhibitor alone on basal AKAR3 ratio, and it did not alter the selective response to PQ-10 in D_2_ MSNs (Fig. 4*B*). These results showed that PP-2A was not involved in the different responsiveness of D_1_/D_2_ and rather suggested the involvement of PP-1.

### PP-1 regulation by DARPP-32 is necessary for the selective responsiveness of D_2_ MSNs to PDE10A inhibition

DARPP-32 is expressed at high levels in both types of MSNs and constitutes a powerful and specific inhibitor of PP-1 when it is phosphorylated at Thr34 (Hemmings et al., 1984). We used a knock-in mutant mouse line in which Thr34 is replaced by an alanine (T34A; Svenningsson et al., 2003). In T34A mice, the effect of PQ-10 on AKAR3 was strongly reduced in D_2_ MSNs (14 ± 2%, *n* = 13, 6 mice), whereas, as in wild-type mice, no effect was observed in D_1_ MSNs (8 ± 1%; Fig. 4*C*,*E*). Normal responses to D_1_ or A_2A_ stimulations were observed at the end of the recording. The phosphatase inhibitor cantharidin unmasked the response to PQ-10 in all MSNs of the DARPP-32 T34A mice (Fig. 4*F*), confirming that, upon inhibition of PP-1, PQ-10 was still capable of increasing the AKAR3 response in these mutant mice. Together, these results show that the inhibition of PP-1 by DARPP-32 is necessary for the difference in responsiveness of D_1_ and D_2_ MSNs.

DARPP-32 also inhibits PKA activity when it is phosphorylated at Thr75 by Cdk5 (Bibb et al., 1999; Nishi et al., 2000). A higher phosphorylation level of this residue in D_1_ MSNs could be responsible for a weaker PKA activity in these neurons. However, in a knock-in mutant mouse line with a Thr75-to-alanine mutation (DARPP-32 T75A; Svenningsson et al., 2003), the profile of the AKAR3 response to PQ-10 was the same as that in wild-type mice (Fig. 4*D*,*E*). Moreover, the Cdk5 inhibitor roscovitine (10 µm) had no effect on the D_1_/D_2_ imbalance in the response to PQ-10 (Fig. 4*G*), ruling out the involvement of Thr75 of DARPP-32 as a critical determinant for the lack of PQ-10-dependent AKAR3 responses in D_1_ MSNs.

### *In vivo* PDE10A inhibition selectively induces histone H3 phosphorylation in D_2_ MSNs of the dorsomedial striatum

**Figure 5.**
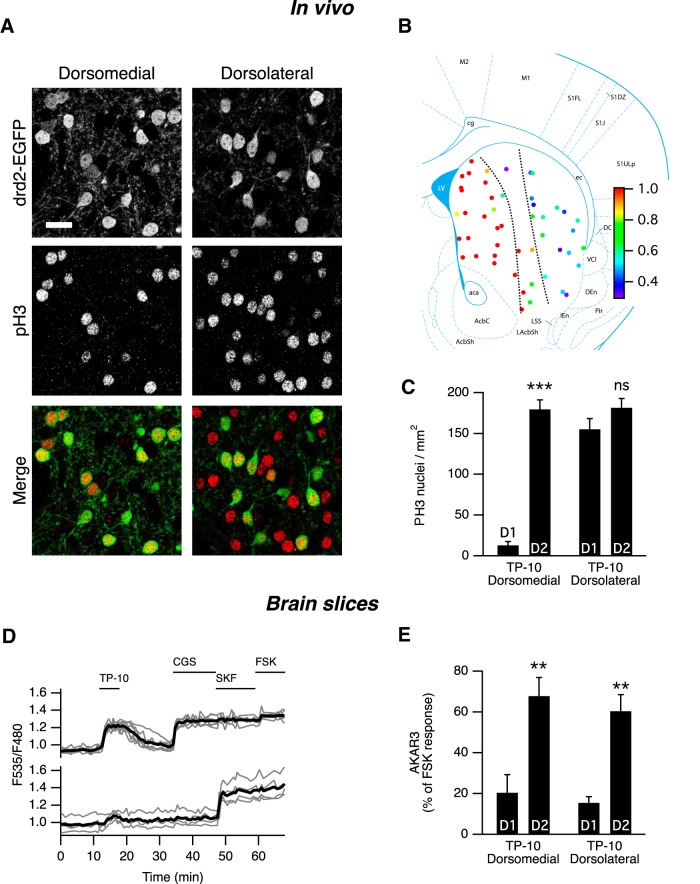
In vivo effects of PDE10A inhibition by TP-10. ***A***, In the medial part of the dorsal striatum of drd2-EGFP adult mice treated with TP-10 (3 mg/kg), PH3 was selectively observed in D_2_ MSNs. In the lateral part of the dorsal striatum, PH3 immunoreactivity was observed in both EGFP-positive and EGFP-negative MSNs. EGFP and PH3 are shown in grayscale, and are overlaid with EGFP in green and PH3 in red (Merge). Scale bar, 20 µm. ***B***, Each color spot represents a position where the relative distribution of D_2_/(D_1_ + D_2_) PH3**-**positive MSNs is indicated in pseudocolor, over a schematic of coronal mouse brain (Franklin and Paxinos, 2007). ***C***, PH3-positive nuclei were quantified in medial and lateral parts of the dorsal striatum as defined by the dotted line in ***B***. The effect of localization was significant (Kruskal–Wallis test followed by **a** Mann–Whitney test with a Dunn–Sidak adjustment test for pairwise multiple comparisons tests, *p* < 10^−4^), with PH3-positive nuclei being preferentially D_2_ MSNs in the medial striatum. ***indicates a difference between EGFP-positive (D_2_) and EGFP-negative (D_1_) MSNs with *p* < 10**^−^** ^4^. ***D***, The preferential AKAR3 response is also observed in the lateral striatum in brain slices from neonate mice. MSNs were transduced for the expression of the AKAR3 biosensor and imaged with wide-field microscope in the lateral striatum. Each trace on the graph indicates the ratio measurement on MSNs expressing AKAR3 and was identified as D_1_ or D_2_ according to their response to either SKF-38393 (SKF, 1 µm) or CGS 21680 (CGS, 1 µm), respectively. The thick black line represents the average of all the traces in each group. TP-10 (100 nm) increased AKAR3 ratio selectively in D_2_ MSNs. ***E***, The same experiment was repeated: there was no effect of localization, and TP-10 increased the AKAR3 ratio selectively in D_2_ MSNs in both the dorsolateral and dorsomedial striatum (two-way ANOVA: localization effect, F_(1,12)_ = 0.374, *p* = 0.374; D_1_/D_2_ effect, F_(1,12)_ = 44.01, *p* < 10**^−^** ^4^; localization × D_1_/D_2_ interaction, F_(1,12)_ = 0.042, *p* = 0.804. Bonferroni’s *post hoc* test: **p < 0.01.). ***C***, ***E***, Error bars indicate the SEM.

Our results showed a marked difference in the responsiveness of D_1_ and D_2_ MSNs to PDE10A inhibitor in slices. We then investigated whether the D_1_/D_2_ imbalance could also be observed *in vivo* by monitoring phospho-histone H3 at Ser10 residue (PH3), a substrate for several protein kinases including PKA (Nowak and Corces, 2004). We used transgenic mice in which D_2_ MSNs are identified by GFP fluorescence (drd2-EGFP mice; Gong et al., 2003; Bertran-Gonzalez et al., 2008) and monitored PH3 by immunofluorescence. In these *in vivo* experiments, we studied the effects of TP-10 (3 mg/kg, i.p.), a PDE10A inhibitor known to produce clear behavioral effects (Schmidt et al., 2008). At this dose, TP-10 induced a large increase in the number of PH3-positive neurons in the striatum 60 min after treatment, compared with vehicle treatment. Quantification showed that 93% of the PH3-immunoreactive neurons were GFP positive (D_2_ MSNs) in the dorsomedial striatum, whereas in lateral parts of the striatum both GFP-negative and GFP-positive D_2_ MSNs exhibited PH3 immunoreactivity (Fig. 5*A–C*). In the *nucleus accumbens*, a sparse and irregular labeling was observed in the shell region, and no immunoreactivity was detected in the core. In all striatal regions, no PH3 immunoreactivity was observed in large neurons expressing low levels of GFP, presumably corresponding to cholinergic interneurons.

Since *in vivo* the selective effects of TP-10 on D_2_ MSNs was observed in the dorsomedial, but not the dorsolateral striatum, we performed a set of experiments in brain slices to compare medial and lateral dorsal striatum using the same inhibitor. The AKAR3 responses to TP-10 (100 nm) were similar, with a strong effect of TP-10 in D_2_ MSNs but not in D_1_ MSNs (Fig. 5*D*,*E*). Altogether, these experiments showed that PDE10A exerts selective effects on D_2_ MSNs in striatal slices and that this selectivity is maintained *in vivo* in the dorsomedial striatum.

### DARPP-32 is required for the *in vivo* effects of TP-10

**Figure 6.**
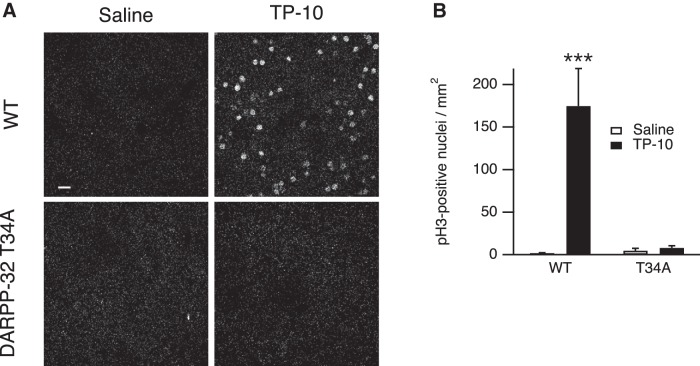
The DARPP-32 T34 residue is required for a TP-10-induced increase of histone H3 phosphorylation in the striatum in adult mice *in vivo*. Wild type (WT) and DARPP-32 T34A mutant mice were treated with TP-10 (3 mg/kg) or vehicle. ***A***, Examples of PH3 immunofluorescence, showing the dramatic reduction of TP-10 effects in the DARPP-32 T34A mutant mice. Scale bar, 20 µm. ***B***, Quantification of the number of PH3-positive neurons in striatal coronal sections. Error bars indicate the SEM. Data were analyzed by a two-way ANOVA: genotype effect, F_(1,12)_ = 13.7, *p* < 0.01; TP-10 effect, F_(1,12)_ = 16.1, *p* < 0.01; genotype × TP-10 interaction, F_(1,12)_ = 14.8, *p* < 0.01. Bonferroni’s *post hoc* test, ***p < 10**^−^** ^3^.

Our experiments in striatal slices showed that the inhibition of PP-1 by DARPP-32 phosphorylated on Thr34 was necessary for the selective responsiveness to PDE10A inhibition on AKAR phosphorylation. We then investigated whether the effects of PDE10A inhibition *in vivo* also depended on the phosphorylation of DARPP-32 at Thr34. T34A knock-in and wild-type littermates were treated with TP-10 (3 mg/kg, i.p.) or vehicle (four animals for each condition) and brain sections analyzed by immunofluorescence for PH3 60 min after injection. Whereas wild-type littermates strongly responded to TP-10, the effect of TP-10 was completely abolished in the DARPP-32 T34A mutant mice in all regions of the striatum (Fig. 6*A*,*B*). These experiments clearly showed that Thr34 in DARPP-32 was required for the effects of PDE10A inhibitor on D_1_ and D_2_ MSNs in both the dorsomedial and dorsolateral striatum *in vivo*.

## Discussion

Our study shows that although PDE10A is expressed and functional in all types of MSNs, its inhibition in striatal slices produces a higher PKA-dependent protein phosphorylation response in D_2_ MSNs than in D_1_ MSNs. We provide evidence that this difference is present in all striatal regions in brain slices and in the dorsomedial striatum of adult mice *in vivo*. Moreover, we show that the regulation of PP-1 activity by DARPP-32 is required for these specific effects of PDE10A inhibitors. These observations provide novel insights into the regulation of the cAMP/PKA pathway in the two populations of MSNs and the possible antipsychotic action of PDE10A inhibitors.

PDE10A is one of the enzymes specifically enriched in the striatum (Seeger et al., 2003; Coskran et al., 2006; Heiman et al., 2008; Lakics et al., 2010; Kelly et al., 2014), and the data reported here show that PDE10A plays an important role in degrading basally produced cAMP in both D_1_ and D_2_ MSNs. MSNs thus contrast with pyramidal neurons of the prefrontal cortex in which basal cAMP is predominantly controlled by PDE4 (Castro et al., 2010). Although PDE4B is also expressed in D_2_ MSNs (Nishi et al., 2008), biosensor imaging of the somatic cytoplasm did not reveal significant effects of its inhibition in MSNs in our conditions.

PDE10A inhibition showed no effect on basal or stimulated cGMP production. A similar lack of effect of PDE10A inhibitors on cGMP has already been reported in brain slice preparations (Nishi et al., 2008). PDE10A thus differs from PDE1 and PDE2, other dual-specificity PDEs expressed in MSNs, which were shown in striatal homogenates to be the major PDEs involved in the control of cGMP levels (Russwurm et al., 2015). Using biosensor-imaging techniques, PDE2 was also shown to be the main PDE that regulated stimulated cGMP, while also regulating cAMP in a cGMP-dependent manner (Polito et al., 2013). *In vivo*, PDE10A inhibition was shown to increase cGMP levels and to affect synaptic transmission (Siuciak et al., 2006; Schmidt et al., 2008; Grauer et al., 2009; Padovan-Neto et al., 2015), and why this effect was not observed in brain slices remains to be determined. One hypothesis to explain this discrepancy might be that, *in vivo*, PDE10A inhibitors recruit nitric oxide synthase-positive striatal interneurons through a global increase in network activity.

**Figure 7.**
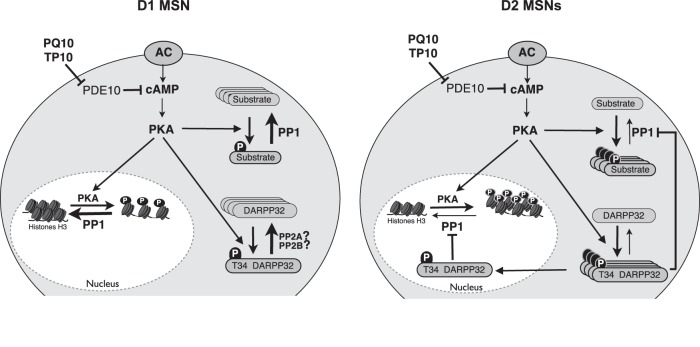
Diagram depicting the D_1_/D_2_ differential response to PDE10A inhibition. PDE10A inhibition increases cAMP and activates PKA to similar levels in D_1_ and D_2_ MSNs. In D_2_ MSNs, DARPP-32 is phosphorylated and inhibits PP-1: PKA substrates thus remain in the phosphorylated state, both in the cytosol and in the nucleus. In D_1_ MSNs, DARPP-32 is in a dephosphorylated state: PP-1 is fully active and dephosphorylates PKA substrates. Differences in PP2A/B activities between D_1_ and D_2_ MSNs may explain this imbalance.

While D_1_ and D_2_ MSNs share a number of cellular features, more detailed studies revealed subtle differences (Valjent et al., 2009), such as different excitability profiles (Gertler et al., 2008; Threlfell et al., 2009). Differences were also reported at the level of PKA-dependent phosphorylation of GABA_A_ receptors and DARPP-32, which were higher in D_2_ MSNs than in D_1_ MSNs (Janssen et al., 2009; Nishi et al., 2008). Our work reveals a possible basis for these D_1_/D_2_ differences, which lies at the level of DARPP-32-mediated PP-1 regulation. In D_1_ MSNs, the Thr34 of DARPP-32 is in a lower phosphorylation state than in D_2_ MSNs and, thus, the PP-1 activity reverts PKA target sites to the dephophorylated state (Fig. 7). This hypothesis implies that a powerful mechanism prevents DARPP-32 from remaining phosphorylated on the Thr34 position selectively in D_1_ MSNs. Thr34 residue is efficiently dephosphorylated by both PP-2A and PP-2B (but not by PP-1; Nishi et al., 1999), and further work is needed to analyze the possible differences in PP2A and PP2B activities between D_1_ and D_2_ MSNs. In contrast to tonic cAMP levels induced by PDE10A inhibition, cAMP signals elicited by D_1_ receptor stimulation lead to a phosphorylation of T34 and inhibition of PP-1 (Bateup et al., 2008), an effect that is also clearly visible on transient responses to dopamine stimulations (Castro et al., 2013). This is consistent with the observation that, in D_1_ MSNs, PDE10A inhibition only affects PKA-dependent modulation of synaptic transmission when cAMP production is stimulated (Mango et al., 2014). This nonlinearity in D_1_ MSNs may improve the detection of powerful but brief events such as the phasic dopamine signal associated with reward and novelty (Schultz, 2010), while filtering out smaller fluctuations in basal cAMP level.

In contrast, in D_2_ MSNs DARPP-32 is phosphorylated on the Thr34 residue, as previously demonstrated (Nishi et al., 2008). In this situation, a moderate cAMP signal, such as that produced by PDE10A inhibition, activates PKA, and, because PP-1 is inhibited, PKA targets remain phosphorylated. Indeed, when DARPP-32 bears the T34A mutation and can no longer inhibit PP-1, D_2_ MSNs fail to respond to PDE10A inhibition (Fig. 7).

Further work is needed to analyze the possible differences in PP-2A and PP-2B activities between D_1_ and D_2_ MSNs that may contribute to the higher level of DARPP-32 phosphorylation on Thr34.

Another potential player in the D_1_/D_2_ differences is the phosphorylation of DARPP-32 on Thr75, which is catalyzed by Cdk5 and is responsible for PKA inhibition (Bibb et al., 1999). Since Thr75 is dephosphorylated by a PKA-activated form of PP-2A containing the B56 subunit (Ahn et al., 2007), it could contribute to a hypersensitive feedforward loop. However, this mechanism did not appear to be critical for PDE10A responses in D_2_ MSNs, since we found no alteration of these responses in DARPP-32 T75A knock-in mutant mice.

The increased responsiveness of D_2_ MSNs at the level of PKA signaling is opposed *in vivo* by the activity of D_2_ receptors: the simple blockade of these receptors by D_2_ antagonists strongly activates cAMP-dependent phosphorylation (Håkansson et al., 2006; Bertran-Gonzalez et al., 2008, 2009; Bonito-Oliva et al., 2011; Valjent et al., 2011). Accordingly, haloperidol or clozapine selectively increases phospho-Thr34-DARPP-32 in D_2_ MSNs, and not in D_1_ MSNs (Bateup et al., 2008). This role of DARPP-32 in D_2_ MSNs is functionally important for some effects of antipsychotic drugs since conditional knockout of DARPP-32 in D_2_ MSNs leads to an increased locomotor activity and a strongly reduced catalepsy upon administration of D_2_ receptor inhibitors (Bateup et al., 2010). Our data show that the inhibition of PDE10A has functional effects that are similar to the blockade of D_2_ receptors since both potentiate the PKA pathway selectively in D_2_ MSNs. The particular sensitivity of D_2_ MSNs includes DARPP-32 Thr34 phosphorylation, which is more increased by PDE10A inhibitors in this population than in D_1_ MSNs (Nishi et al., 2008). Thus, hypersensitivity of DARPP-32 Thr34 phosphorylation could be a critical factor to account for the responsiveness of D_2_ MSNs to the blockade of either D_2_ receptors or PDE10A activity.

The cellular effects of PDE10A inhibition affect MSNs neuronal properties, which are integrated through the basal ganglia network. For example, PDE10A inhibition was shown to potentiate D-amphetamine-dependent dopaminergic neuromodulation *in vivo* (Sotty et al., 2009). In addition, PDE10A inhibition was shown to massively increase cGMP levels *in vivo* (Siuciak et al., 2006; Schmidt et al., 2008; Grauer et al., 2009), whereas, no effect was observed in brain slice preparations (Nishi et al., 2008; this study). This discrepancy possibly results from the recruitment of striatal NOergic interneurons through network activity resulting indirectly from PDE10A inhibition.

*In vivo*, the inhibition of PDE10A also selectively activates D_2_ MSNs in the medial striatum. Interestingly, this striatal subregion is innervated by prefrontal and cingulate cortices, which are involved in the limbic system, and dopamine neurons originating from the ventral tegmental area. D_1_ and D_2_ receptors exert contrasting roles selectively in the dorsomedial striatum during behavioral inhibition in the stop-signal task in rats (Eagle et al., 2011), and lesions of the dorsomedial striatum disrupt prepulse inhibition (Baldan Ramsey et al., 2011). This region is also involved in early motor learning and is required for the cataleptic effects of haloperidol and for amphetamine motor response sensitization (Durieux et al., 2012). Medial striatum thus appears as a limbic system-related region that could be affected in schizophrenia.

The differences observed between medial and lateral striatum likely involve network effects and differences in synaptic plasticity reported between these two striatal subregions (Lovinger, 2010), and further work should precisely show how these spatial differences are related to behavior. Our work nonetheless shows that the D_1_/D_2_ difference is present in brain slices in both medial and lateral striatum, and remains when network activity is blocked, showing that the D_1_/D_2_ difference is an intrinsic property of D_1_ and D_2_ MSNs. The *in vivo* effects of PDE10A inhibitors were totally abolished in T34-DARPP-32 mutant mice, confirming the role of the DARPP-32/PP-1 loop as the initial determinant of the positive PKA response obtained during PDE10A inhibition.

Further work is needed to determine whether the imbalance in PKA signal integration between D_1_ and D_2_ MSNs might be of interest to understand the pathophysiology of other diseases that affect neuromodulatory processes in basal ganglia such as Parkinson’s disease or Huntington’s disease (Threlfell and West, 2013).

## Synthesis

The decision was a result of the Reviewing Editor Quentin Pittman and the peer reviewers coming together and discussing their recommendations until a consensus was reached. A fact-based synthesis statement explaining their decision and outlining what is needed to prepare a revision is listed below. The following reviewers agreed to reveal their identity: Sean Smith, Han-Ting Zhang

Synthesis:

While there are a number of editorial and presentation issues that must be addressed, it seems that the most important issue is the point raised by reviewer 1 concerning the cGMP data and the possibility that you are outside the dynamic range of your sensor. This point must be addressed or additional data provided.

Reviewer 1

The authors submitted a well written manuscript describing the impact of PDE10A inhibitors on cAMP and cGMP signaling in distinct populations of striatal neurons. The authors performed several well thought out experiments to investigate the signaling cascades downstream of cAMP in D1 and D2 neurons using a powerful imaging technique.

Major Comment:

I have a significant concern about the reliability of the cGMP data in this experiment. There have been several publications that clearly show administration PDE10A inhibitors significantly increase cGMP in striatal tissue using immunoassay based techniques. The data the authors present using the cygnet2 bio sensors is not convincing as the it appears the signal from the NO donor DENO maxes out the dynamic range of the sensors. I would suggest the authors use an additional technique, such as an immunoassay to clearly demonstrate that PDE10A inhibitors do not increase cGMP in their slices.

Minor Comment

There are several typing errors in the manuscript including paragraph two of the discussion

Reviewer 2

This study examines the effects of PDE10A inhibition in vivo by immunohistochemistry, and imaged cAMP, PKA and cGMP signals with biosensors in mouse brain slices. The results show that D1 and D2 MSNs differ in the integration of cAMP signal due to greater inhibition of PP-1 by DARPP-32 in D2 MSNs than in D1, suggesting that PDE10A inhibitors share with antipsychotics the property of preferentially activating PKA-dependent signaling in D2-MSNs. The experiments are well designed and performed, data are appropriately analyzed and presented, and the manuscript is well written. I only have a few issues that need to be addressed.

1. It would help understand the study better if an illustration is provided to show the signals interacting with PDE10A.

2. It is not clear why TP-10 is switched in the last two experiments (histone H3 phosphorylation and DARPP-32). It would make more sense if PQ-10 is used throughout the experiments.

3. It is necessary to specify whether one-way or two-way ANOVA was used for the data analyses.

4. A bar graph panel is needed to show the effect of PQ-10 alone on AKAR3, which would be in contrast to the effect in the presence of quinpirole (Fig. 3B).

Reviewer 1

The authors submitted a well written manuscript describing the impact of PDE10A inhibitors on cAMP and cGMP signaling in distinct populations of striatal neurons. The authors performed several well thought out experiments to investigate the signaling cascades downstream of cAMP in D1 and D2 neurons using a powerful imaging technique.

- We would like to thank the Reviewer for his in depth analysis of our manuscript and his positive comments.

Major Comment:

I have a significant concern about the reliability of the cGMP data in this experiment. There have been several publications that clearly show administration PDE10A inhibitors significantly increase cGMP in striatal tissue using immunoassay based techniques. The data the authors present using the cygnet2 biosensors is not convincing as the it appears the signal from the NO donor DENO maxes out the dynamic range of the sensors. I would suggest the authors use an additional technique, such as an immunoassay to clearly demonstrate that PDE10A inhibitors do not increase cGMP in their slices.

- Based on the literature, we indeed expected a strong effect of PDE10A inhibitors on cGMP levels, but surprisingly, our recordings using the cygnet2 biosensor did not reveal any effect. While performing these experiments, however, we came across the strong effect of PDE10A inhibitors on the cAMP/PKA signaling cascade and the unsuspected difference between D1 and D2 neurons, which became the main focus of our manuscript.

- We understand that our presentation of the cGMP results was too brief and we have now improved the figure and statistics. Figure 3F now illustrates a typical experiment where DEANO increased the ratio to a steady-state level. The final application of IBMX further increased the ratio level, an effect which was qualitatively visible on the ratio traces. This effect of IBMX was statistically significant (Figure 3G), which shows that the biosensor was not saturated. However, in these conditions, PQ-10 showed no effect. The same protocol had been previously used on the same preparation, which revealed the implication of PDE2 in the regulation of cGMP (Polito et al, 2013). This suggests that PDE2 action on cGMP overpowers any potential contribution of PE10A in the regulation of NO-induced cGMP. A citation of Russwurm 2015 is now discussed in more detailed. Further work would be needed to identify a situation where PDE10A might possibly show an effect on cGMP, but we think that this question is not in the scope of our manuscript.

Minor Comment

There are several typing errors in the manuscript including paragraph two of the discussion

- We corrected two typos in the 2nd paragraph of the discussion.

Reviewer 2

This study examines the effects of PDE10A inhibition in vivo by immunohistochemistry, and imaged cAMP, PKA and cGMP signals with biosensors in mouse brain slices. The results show that D1 and D2 MSNs differ in the integration of cAMP signal due to greater inhibition of PP-1 by DARPP-32 in D2 MSNs than in D1, suggesting that PDE10A inhibitors share with antipsychotics the property of preferentially activating PKA-dependent signaling in D2-MSNs. The experiments are well designed and performed, data are appropriately analyzed and presented, and the manuscript is well written. I only have a few issues that need to be addressed.

- We are very grateful to the Reviewer for his detailed analysis of our manuscript and his positive comments.

1. It would help understand the study better if an illustration is provided to show the signals interacting with PDE10A.

- A 7th figure is added in the revised manuscript.

2. It is not clear why TP-10 is switched in the last two experiments (histone H3 phosphorylation and DARPP-32). It would make more sense if PQ-10 is used throughout the experiments.

- PQ-10, TP-10 and MP-10 are not commercially available: we first had access to PQ-10, and only at a later stage obtained TP-10. In any case, these inhibitors were tested in brain slices with similar effects on PKA activity (Figure 3B-C, 5E). For the in vivo studies we used TP-10 because of its extensive and well documented effects in living animals. (Schmidt et al., 2008, J Pharmacol Exp Ther, 325, 681-90). Thus, we could select an appropriate dose which exhibited biochemical and behavioral effects.

3. It is necessary to specify whether one-way or two-way ANOVA was used for the data analyses.

- This information is now indicated in the figure legends.

4. A bar graph panel is needed to show the effect of PQ-10 alone on AKAR3, which would be in contrast to the effect in the presence of quinpirole (Fig. 3B).

- This bar graph has been added for visual comparison.
